# Cognitive Impairment Among Older Adults With Diabetes Mellitus in Puducherry: A Community-Based Cross-Sectional Study

**DOI:** 10.7759/cureus.12488

**Published:** 2021-01-04

**Authors:** Manimozhi Subramanian, Kavita Vasudevan, Anandaraj Rajagopal

**Affiliations:** 1 Department of Community Medicine, Indira Gandhi Medical College & Research Institute, Puducherry, IND

**Keywords:** cognitive impairment, diabetes mellitus, older adults, hmse

## Abstract

Background

Cognitive impairment is a global public health problem in the elderly population. There is increasing evidence that diabetes mellitus predisposes to cognitive impairment. Early diagnosis and management of cognitive impairment can delay the onset of dementia, thereby improving self-care and quality of life of diabetic patients. This study intends to assess cognitive impairment, and the factors influencing cognitive impairment among older adults with diabetes mellitus in Puducherry.

Methods

A community-based cross-sectional study was conducted in field practice areas of a Government Medical College in Puducherry between April and June 2019. After obtaining ethical approval, 240 registered diabetic patients aged 55 years and above were randomly selected. Data on demographic profile and clinical variables were collected using a semi-structured questionnaire. Cognitive function was assessed using the Hindi Mental State Examination (HMSE) tool, and participants who scored below 26 were considered to have cognitive impairment.

Results

Among 240 participants, 67.9% were aged 60 years and above, 62.5% were females, and 83.8% were unemployed. The proportion of cognitive impairment among older adults with diabetes was 30.0% (95% confidence interval (CI): 24.5-36.03). The mean ± standard deviation of the HMSE Score was 26.13 ± 3.8, and the median score was 27. Female gender (P= 0.02, adjusted prevalence ratio (aPR) = 5.31, 95% CI: 1.34-21), widowhood status (P= 0.005, aPR= 2.71, 95% CI: 1.34-5.46), illiteracy (P<0.001, aPR= 3.55, 95% CI: 1.78-7.07), and presence of probable symptomatic hypoglycemia (P=0.02, aPR= 2.18, 95% CI: 1.13-4.20) were significant predictors of cognitive impairment in the study population by multivariate analysis.

Conclusion

Almost one-third of older adults with diabetes were found to be at risk of cognitive impairment. Older diabetic patients with identified risk factors may be prioritized for a screening of cognitive impairment at the primary care level.

## Introduction

Dementia is a syndrome where there is a deterioration of cognitive function. It affects memory, thinking, behavior, and ability to perform daily activities. Dementia has a physical, psychological, and economic impact, not only on people with dementia but also on their caregivers and society. Although dementia is more common in older people, it is not a normal part of aging. It is estimated that around 50 million people have dementia worldwide, with nearly 60% living in developing countries [1]. The total number is projected to increase to 82 million cases by 2030 and 152 million cases by 2050, with the majority of them living in low- and middle-income countries [1]. Since there is no treatment available currently to cure dementia, early diagnosis to identify the underlying cause and treating the accompanying illness becomes an important goal for dementia care.

As per the International Diabetes Federation, 463 million adults are currently living with diabetes globally, and it is projected to rise to 700 million by 2045 [2]. One in five people with diabetes are above 65 years of age, and 10% of global expenditure is spent on diabetes [2]. Diabetes mellitus (DM) affects all systems of the body, including the brain leading to cognitive dysfunction. There is increasing evidence that diabetes predisposes to cognitive decline leading to dementia in animal models and humans [3,4]. Although macro-vascular and micro-vascular complications of diabetes are well recognized, there is less awareness regarding other conditions such as cognitive dysfunction and depression. Assessing cognitive function in a diabetic is important because of its impact on self-care practices and quality of life [5]. Also, there is a paucity of data on cognitive impairment among people with diabetes in Puducherry. So, this study intended to assess cognitive impairment and the factors influencing cognitive impairment among the study participants.

This article was previously presented as a poster at a conference (Poster: Manimozhi S, Kavita V, Anandaraj. Cognitive Impairment Among Older Adults With Diabetes Mellitus in Puducherry: A Community-Based Cross-Sectional Study. KOVAICON; September 6, 2019).

## Materials and methods

This community-based descriptive cross-sectional study was conducted between April and June 2019, in the field practice areas of a Government Medical College in Puducherry. The study protocol was approved by the Institute Research Committee and Institute Ethics committee.

Diabetic patients of either gender, aged 55 years and above, registered under non-communicable disease (NCD) clinic in selected health centers were included in the study. Participants who were a known case of neuropsychiatric disorders or on psychoactive drug use and participants who were unable to communicate and follow the investigator's instructions were excluded from the study.

Presuming the prevalence of cognitive impairment among the older people with diabetes as 63.3% based on a previous study [6], the sample size required at 95% confidence limit, 6.5% absolute precision, and a 10% non-response rate was calculated as 234. A total of 240 study participants, 120 from the Urban Health Training Centre and 120 from the Rural Health Training Centre, were selected.

As the pensionable age in Puducherry is 55 years, participants aged 55 years or above were considered as older adults [7,8]. One hundred and fifty minutes per week or 30 minutes per day for at least five days in a week of moderate-intensity aerobic physical activity was considered as adequate physical activity [9]. The presence of one or more symptoms of hypoglycemia, which gets relieved on taking oral carbohydrates without a test of plasma glucose, in the last 30 days, was the operational definition for probable symptomatic hypoglycemia [10]. For alcohol and tobacco use, the participants were categorized into current, ever, and never users. Current tobacco users were those who consumed tobacco in any form in the last 30 days. Current users of alcohol were those who consumed alcohol in any amount in the last 12 months. The participants who had fasting blood sugar >125 mg/dl and post-prandial blood sugar/random blood sugar >200 mg/dl were considered to have uncontrolled diabetes [11]. The body mass index (BMI) criteria for Asians by the regional office for the western pacific region of WHO was used to define underweight (< 18.5 kg/m2), overweight (23 to 24.9 kg/m2), and obesity ( ≥ 25 kg/m2) among the study participants.

Eligible study participants were randomly selected from the NCD register available in the facility. A home visit was made with the help of a health assistant, auxiliary nurse midwife (ANM), or accredited social health activist (ASHA). After obtaining written informed consent from the study participants, a pre-tested semi-structured questionnaire was used to collect information on the socio-demographic profile and clinical variables. Participants were screened for cognitive impairment using Hindi Mental State Examination (HMSE) tool. Items in the questionnaire were translated forward (English to Tamil) and backward (Tamil to English) by the investigator proficient in both languages. Content validation was done by two public health experts and a psychiatrist.

Hindi Mental State Examination tool was developed by the Indo-US Cross-National Dementia Epidemiology Study. Though the Mini Mental State Examination (MMSE) is the preferred tool for assessing cognitive function, it has few limitations. For the use of the MMSE tool, permission is required, and it can be administered only if the participant is literate [12]. Hence HMSE tool was developed by modifying the Mini Mental State Examination tool and was validated for use in a largely illiterate rural elderly population in India [12]. This tool consists of simple questions and a task, with a maximum score of 30. Participants with a score of less than 26 are considered to have cognitive impairment. They were further classified into mild (score 21-25), moderate (score 11 to 20), or severe (score ≤ 10) based on their scores [13]. Participants with lower scores were referred to a higher center for neuropsychiatric evaluation and further management.

Data were entered in a Microsoft Excel sheet (Microsoft® Corp., Redmond, WA), and data analysis was done using the Statistical Package for Social Sciences (SPSS) software for Windows, version 22.0 (IBM Corp., Armonk, NY). Categorical variables were described as number and percentage, whereas continuous variables were described as mean ± standard deviation (SD). A Chi-square test was applied to identify the association between each independent variable and cognitive impairment. A P*-*value of <0.05 was considered statistically significant. The variables that had a significant association in bivariate analysis were entered into the logistic regression model to identify the predictor variables of cognitive impairment. The magnitude of association was presented as an adjusted prevalence ratio with 95% confidence intervals.

## Results

Among 240 study participants, 163 (67.9%) were aged 60 years and above. The mean age (±SD) of the study subjects was 63.9 ± 7.1 years. More than one third (38.7%) of the study participants were illiterate, 62.5% were females, and 83.7% were unemployed.

The mean (±SD) of the HMSE Score was 26.13 ± 3.8, and the median HMSE score was 27. The mean HMSE score was 24.2 ± 3.8 for illiterate, 26.1 ± 2.9 for primary level of education, 27.8 ± 4.2 for middle school, and 29.1 ± 1.5 for high school and above.

The proportion of cognitive impairment among older adults with diabetes was 30.0% (95% CI: 24.5-36.03). Out of 240 study subjects assessed using the HMSE tool, 40 (16.7%) study participants had mild cognitive impairment, 31 (12.9%) had moderate, and one participant (0.4%) had severe cognitive impairment (Table 1).

**Table 1:** Distribution of demographic variables and the proportion of cognitive impairment among the study participants (n=240)

Variable		Frequency (n=240) (%)	Cognitive impairment			
			Mild (n=40)	Moderate (n=31)	Severe (n=01)	Total (n=72) (%)
Residence	Urban	120 (50.0)	21	15	01	37 (51.4)
	Rural	120 (50.0)	19	16	-	35 (48.6)
Age	55-59	77 (32.1)	05	08	-	13 (18.1)
	60-69	96 (40.0)	20	11	-	31 (43.1)
	70-79	58 (24.2)	12	09	-	21 (29.2)
	80-85	9 (3.7)	03	03	01	07 (9.7)
Gender	Male	90 (37.5)	06	03	-	09 (12.5)
	Female	150 (62.5)	34	28	01	63 (87.5)
Marital status	Married	160 (66.7)	17	12	-	29 (40.3)
	Widow/widower	80 (33.3)	23	19	01	43 (59.7)
Type of family	Nuclear	126 (52.5)	20	17	-	37(51.4)
	Joint	114 (47.5)	20	14	01	35 (48.6)
Education	Illiterate	93 (38.7)	23	25	-	48 (66.7)
	Primary	64 (26.7)	13	06	-	19 (26.4)
	Middle	47 (19.6)	02	-	01	03 (4.2)
	High school & Above	36 (15.0)	02	-	-	02 (2.8)
Employment	Unemployed	201 (83.8)	35	31	1	67 (93.1)
	Employed	39 (16.2)	05	-	-	05 (6.9)

Among the respondents, the co-morbidities reported were hypertension (n= 138, 57.5%), coronary artery disease (n=24, 10.0%), dyslipidemia (n= 12, 5.0%), bronchial asthma (n= 10, 4.2%), one old case of pulmonary tuberculosis and one participant was on antiretroviral therapy for HIV. All study subjects had type 2 DM, of which 54.6% had uncontrolled sugar levels. The majority of the study participants (90.4%) were on treatment with oral hypoglycemic agents (OHA). Six participants were on insulin, and 17 (7.1%) were on treatment with both insulin and OHA. The ratio of cognitive impairment was higher among the participants who were on treatment with insulin when compared to those on OHA. Among the eight (3.3%) participants who had a BMI of less than 18.5, seven had cognitive impairment. One hundred sixteen (48.3%) participants had a BMI of more than 25, of which 31 participants had impaired cognition (Table 2).

**Table 2:** Distribution of clinical variables and the proportion of cognitive impairment among the study participants

Variable		Frequency (n=240) (%)	Cognitive impairment			
			Mild (n=40)	Moderate (n=31)	Severe (n=01)	Total (n=72) (%)
Duration of illness	< 5 years	113 (47.1)	18	15	-	33 (45.8)
	5-9 years	64 (26.6)	10	12	-	22 (30.6)
	≥ 10 years	63 (26.3)	12	4	01	17 (23.6)
Co-morbidity	Present	153 (63.8)	26	24	-	50 (69.4)
	Absent	87 (36.2)	14	07	01	22 (30.6)
Treatment	Oral Hypoglycemic Agents	217 (90.4)	33	30	-	63 (87.5)
	Insulin	06 (2.5)	04	-	01	05 (6.9)
	Both	17 (7.1)	03	01	-	04 (5.6)
Probable symptomatic hypoglycemia	Present	95 (39.6)	20	18	01	39 (54.2)
	Absent	145 (60.4)	20	13	-	33 (45.8)
Blood sugar	Under control	109 (45.4)	18	13	01	32 (44.4)
	Uncontrolled	131 (54.6)	22	18	-	40 (55.6)
Exercise	Adequate	59 (24.6)	12	04	-	16 (22.2)
	Inadequate	181 (75.4)	28	27	01	56 (77.8)
Body mass index	< 18.5	08 (3.3)	01	05	01	07 (9.7)
	18.5-22.9	66 (27.5)	14	04	-	18 (25.0)
	23-24.9	50 (20.8)	10	06	-	16 (22.2)
	25-29.9	83 (34.6)	11	14	-	25 (34.8)
	≥ 30	33 (13.8)	04	02	-	06 (8.3)

There was no significant difference in the proportion of cognitive impairment between the urban (30.83%) and rural (29.17%) study subjects (Prevalence ratio= 1.06, 95% CI: 0.62-1.88, P= 0.77). Increasing age, female gender, illiteracy, and unemployment had a significant association with cognitive impairment in bivariate analysis (Table 3).

**Table 3:** Association of demographic variables with cognitive impairment (n=240) *significant P-value, ref: reference variable

Variable		Frequency n	Cognitive impairment n (%)	Prevalence ratio (95% Confidence Interval)	P-value
Residence	Urban	120	37 (30.8)	1.06 (0.72- 1.56)	0.778
	Rural	120	35 (29.2)	1.00 (ref)	
Age	≥ 60 years	163	59 (36.2)	2.14 (1.25- 3.67)	0.002*
	<60 years	77	13 (16.9)	1.00 (ref)	
Gender	Female	150	63 (42.0)	4.20 (2.20- 8.03)	<0.001*
	Male	90	9 (10.0)	1.00 (ref)	
Marital status	Widow	80	43 (53.8)	2.97 (2.01- 4.4)	<0.001*
	Married	160	29 (18.1)	1.00 (ref)	
Education	Illiterate	93	48 (51.6)	3.16 (2.09- 4.79)	<0.001*
	Literate	147	24 (16.3)	1.00 (ref)	
Employment	Unemployed	201	67 (33.3)	2.60 (1.12- 6.03)	0.011*
	Employed	39	5 (12.8)	1.00 (ref)	
Family type	Joint	114	35 (30.7)	1.05 (0.71- 1.54)	0.821
	Nuclear	126	37 (29.4)	1.00 (ref)	

Twenty-eight (11.6%) study participants were current users of tobacco, and 22 (9.2%) study participants were current users of alcohol. Probable symptomatic hypoglycemia and alcohol use had a significant association with cognitive impairment by bivariate analysis. There was no significant association between duration of diabetes and cognitive impairment (Table 4).

**Table 4:** Association of clinical variables with cognitive impairment (n=240) *significant P-value, ref: reference variable, OHA: oral hypoglycemic agents

Variable		Frequency n	Cognitive Impairment present n (%)	Prevalence ratio (95% Confidence Interval)	P-value
Co-morbidity	Present	153	50 (32.7)	1.29 (0.84-1.98)	0.230
	Absent	87	22 (25.3)	1.00 (ref)	
Duration of illness	< 5 years	113	33 (29.2)	1.05 (0.71- 1.55)	0.800
	≥ 5 years	127	39 (30.7)	1.00 (ref)	
Treatment with	Insulin	23	9 (39.1)	1.35 (0.78- 2.34)	0.315
	OHA	217	63 (29.0)	1.00 (ref)	
Probable symptomatic hypoglycemia	Present	95	39 (41.1)	1.80 (1.22- 2.65)	0.002*
	Absent	145	33 (22.8)	1.00 (ref)	
Tobacco users	Ever	56	19 (33.9)	1.18 (0.77- 1.81)	0.464
	Never	184	53 (28.8)	1.00 (ref)	
Alcohol users	Ever	54	9 (16.7)	0.49 (0.26- 0.92)	0.015*
	Never	186	63 (33.9)	1.00 (ref)	
Physical activity	Inadequate	181	56 (30.9)	1.14 (0.71- 1.83)	0.578
	Adequate	59	16 (27.1)	1.00 (ref)	
Blood sugar levels	Uncontrolled	131	40 (30.5)	1.04 (0.70- 1.53)	0.843
	Under control	109	32 (29.4)	1.00 (ref)	
Body mass index	Malnourished	174	54 (31.0)	1.06 (0.71- 1.60)	0.570
	Normal	66	18 (27.3)	1.00 (ref)	

The variables which had a significant association in bivariate analysis were entered into the logistic regression model. Female gender, widowhood status, illiteracy, and the presence of probable symptomatic hypoglycemia were found to be significant predictors of cognitive impairment in multivariate analysis (Table 5).

**Table 5:** Predictor variables of cognitive impairment on logistic regression analysis

Variable	Adjusted Prevalence Ratio	95% Confidence interval	P value
Age ≥ 60 years	1.96	0.88- 4.35	0.098
Female gender	5.31	1.34- 21	0.017
Widowhood status	2.71	1.34- 5.46	0.005
Illiterate	3.55	1.78- 7.07	<0.001
Unemployed	0.69	0.18- 2.59	0.585
Presence of probable symptomatic hypoglycemia	2.18	1.13- 4.20	0.019
Alcohol use	2.62	0.66- 10.43	0.170

## Discussion

This was a community-based study conducted in the field practice area of the Urban and Rural Health Training Centres of a Government Medical College in Puducherry. The proportion of cognitive impairment in the present study was 30.0%. Other studies reported varied prevalence, 9.6% by Tiwari et al., 10.8% by Krishnamoorthy et al., 33.7% by Khullar et al., 42% by Mukherjee et al., 74% by Pednekar et al. [14-18]. This difference in proportion was probably due to variation in the study population, study tool, and different cut-off scores used for defining the cognitive impairment.

The proportion of cognitive impairment among the study participants below 60 years was 16.8%, between 60 and 80 years was 33.5%, and 80 years or above was 87.5%. The proportion of cognitive impairment in the present study significantly increased with increasing age in bivariate analysis. Research evidence suggests that this association with increasing age might be due to a decline in volume and integrity of white matter and its tract [19].

In the present study, female gender had a significant association with cognitive impairment. Studies have reported that female gender was an independent risk factor for neurocognitive impairment and women with impaired fasting glucose had poor cognitive scores compared to those with normal glucose [16,20]. Genetic predisposition of females due to the presence of apolipoprotein E4 allele, brain-derived neurotrophic factor Met 66 allele, estrogen, the difference in the occupation or educational level in women are probable reasons for the significant association of female gender with cognitive impairment [21].

Widowhood status was found to be a significant predictor of cognitive impairment in the present study. The loss of a spouse is one of the most stressful events for older people. Stress can induce increased glucocorticoid secretion resulting in hippocampal atrophy, resulting in cognitive dysfunction in these individuals [22]. But its association with cognitive impairment in a diabetic individual needs further research.

In this study, illiteracy had a significant association with cognitive impairment. The mean HMSE score was higher in literate than illiterate. Literature states that illiteracy is an independent risk factor for dementia. The high prevalence of dementia in illiterates may be due to poor adaptation to neuropsychological tests and low cognitive reserve [13]. There was a significant association of unemployment with cognitive impairment at the bivariate level, but there was no significant association at the multivariate level. Unemployed elderly might be less physically active than their employed counterparts. Exercise has a direct effect on preserving neurogenesis and favoring neuroplasticity [23]. Studies have reported an association of physical exercise with a decreased risk of cognitive impairment. But these associations in diabetic individuals need to be explored in future research. The present study could not observe a favorable association of physical activity with cognitive impairment. There was no association between the duration of diabetes and cognitive impairment, which was similar to the results obtained in a study by Mukherjee et al. [17].

In the current study, there is a significant association between probable symptomatic hypoglycemia and cognitive impairment. Studies show there is a bidirectional relationship between hypoglycemia and dementia. Acute severe hypoglycemic episodes can lead to chronic subclinical brain damage, cognitive decline, and subsequent dementia [24]. Hyperglycemia mediated advanced glycosylated end-product production, and oxidative stresses can damage neurons and vascular endothelium leading to cognitive dysfunction [5]. Hence strict control of blood sugar plays an important role in the onset of cognitive decline among diabetic patients.

A community-based study using a validated tool appropriate for the local population is the major strength of the study. But HbA1C and blood sugar levels could not be tested due to limited resources. Instead, the latest blood sugar value, preferably tested within the last three months, was considered for knowing whether diabetes is under control. Further, HMSE is a screening tool, and patients with a lower score need further neuropsychiatric evaluation to know the underlying cause and further management.

## Conclusions

Almost one-third of older adults with diabetes were found to be at risk of cognitive impairment. Female gender, widowed status, illiteracy, and the presence of probable symptomatic hypoglycemia were found to be significant predictors of cognitive impairment in the diabetic population. Study participants with attributes identified as significant in the current study may be prioritized for the screening of cognitive impairment at the primary care level. Policy-makers should integrate the screening of cognitive impairment along with the National Programme for Prevention and Control of Cancer, Diabetes, Cardiovascular Diseases and Stroke (NPCDCS) and also train primary care physicians for screening and providing comprehensive care to individuals with cognitive impairment.
